# Bacteriophage genotyping using BOXA repetitive-PCR

**DOI:** 10.1186/s12866-020-01770-2

**Published:** 2020-06-11

**Authors:** Dragica Damnjanovic, Xabier Vázquez-Campos, Daniel L. Winter, Melissa Harvey, Wallace J. Bridge

**Affiliations:** School of Biotechnology and Biomolecular Sciences, Faculty of Science, UNSW Sydney, Kensington, Australia

**Keywords:** Bacteriophage, Phage genotyping, Repetitive-PCR

## Abstract

**Background:**

Repetitive-PCR (rep-PCR) using BOXA1R and BOXA2R as single primers was investigated for its potential to genotype bacteriophage. Previously, this technique has been primarily used for the discrimination of bacterial strains. Reproducible DNA fingerprint patterns for various phage types were generated using either of the two primers.

**Results:**

The similarity index of replicates ranged from 89.4–100% for BOXA2R-PCR, and from 90 to 100% for BOXA1R-PCR. The method of DNA isolation (*p* = 0.08) and the phage propagation conditions at two different temperatures (*p* = 0.527) had no significant influence on generated patterns. Rep-PCR amplification products were generated from different templates including purified phage DNA, phage lysates and phage plaques. The use of this method enabled comparisons of phage genetic profiles to establish their similarity to related or unrelated phages and their bacterial hosts.

**Conclusion:**

The findings suggest that repetitive-PCR could be used as a rapid and inexpensive method to preliminary screen phage isolates prior to their selection for more comprehensive studies. The adoption of this rapid, simple and reproducible technique could facilitate preliminary characterisation of a large number of phage isolates and the investigation of genetic relationship between phage genotypes.

## Background

Repetitive DNA sequences constitute a substantial component of both eukaryotic and prokaryotic genomes. In some higher plant species, they can account for up to 90% of the genomic DNA [1], while in humans DNA repeats comprise nearly half of the genome [2]. The presence or absence of certain types of repeats, diversity in their nucleotide sequences, their size, location and copy number per genome characterize various bacterial species, even those with the smallest genomes [3]. Interspersed repeats play a significant role in genomic rearrangements, such as inversions, deletions, duplications and translocations [3]. The proposed functional roles of repetitive sequences involve the regulation of coding sequence expression and the formatting necessary for genome packaging; DNA repair and restructuring; genome replication and transmission to progeny cells; formation of nucleoprotein complexes; and formation of a characteristic genome system organization that allows for evolutionary significant changes without altering coding sequences [4].

Specific families of interspersed DNA elements have been observed in many bacterial and archaeal genomes [3, 5], while bacteriophages are considered to carry few repetitive elements [5]. The BOX family of repetitive DNA elements, consisting of different combinations of three sequence sub-motifs, boxA, boxB, and boxC, was originally identified in Gram-positive *Streptococcus pneumoniae* [6]. Hybridization studies have shown that only boxA sequences are highly evolutionary conserved. The outwardly facing repetitive primers BOXA1, BOXA1R and BOXA2R that are complementary to the consensus sequences of boxA, when used as single primers in the repetitive-PCR, generated complex fingerprint patterns in various Gram-positive and Gram-negative bacterial species [7]. BOXA-based primers have since been used for genotyping diverse bacterial species in various ecological [8], epidemiological [9] and industrial application studies [10].

The optimal performance of dairy starter cultures is challenged by the risk of lytic bacteriophage (phage) infection [11, 12] and indeed, phage may pose a problem to any industry based on bacterial fermentation [13]. In recent years, there has been a renewed interest in phage from the perspective of their beneficial applications, such as phage therapy for treating pathogens [14, 15]. Of great utility for the dairy industry are multiplex PCR systems that can detect and classify the three main lactococcal phage species, 936, P335 and c2, as well as *Streptococcus thermophilus* and *Lactobacillus delbrueckii* phages [16, 17]. These rapid and sensitive tests are based on the generation of specific PCR amplification products for each phage species; however, they do not enable the identification of individual phage strains. Methods that can be applied for genetic characterization of phages involve restriction digestion of genomic DNA [18]; multilocus sequence typing (MLST) [19]; restriction fragment length polymorphism (RFLP) or a denaturing gradient gel electrophoresis (DGGE) of a particular gene [20]; random amplification of polymorphic DNA (RAPD)-PCR [18, 21, 22] and genomic sequencing [23]. However, these methods are not necessarily the most suitable for routine use due to time or cost-associated constraints**.** MLST and DGGE require a priori genetic information [21]. Restriction digestion of genomic DNA and DNA/DNA hybridizations are considered time-consuming and often require large quantities (μg) of pure DNA [21]. Additionally, the genomes of some phages can be resistant to restriction enzymes, which may be due to a scarcity of cleavage sites [24]; a base modification within the recognition sequence, genome methylation or other antirestriction mechanisms [18]. This imposes the need to use several restriction enzymes to ensure digestion [18]. While MLST can distinguish phages with the same RFLP pattern [19], it may not be universally applicable for fingerprinting all phage types. For example, it has proved suitable for phylogenetic analysis of the 936-like phages of *Lactococcus lactis*, but could not be used to generate amplification products from the c2 and P335 phage groups [19]. Whole-genome phage sequencing is associated with substantial cost and technical difficulties [22], and may be impractical for routine use.

RAPD-PCR and rep-PCR are in the category of fast and inexpensive genotyping methods, but RAPD-PCR has been reported to have poor reproducibility. This is due to the use of short and arbitrary primers which target randomly distributed sequences [25] and thus requires substantial optimization of conditions [18].

Our preliminary work on genetic characterization of *Lactococcus lactis* phages showed that restriction digestion was not successful for all of them rendering comprehensive phage comparison unachievable (unpublished data). This prompted our search for an alternative method for phage differentiation. To this end, repetitive-PCR using BOXA-based primers, which we have previously successfully applied to discriminate bacterial and yeast isolates [26] was explored. The aim of this study was to investigate whether repetitive-PCR typing is applicable to bacteriophage, by determining whether amplified viral DNA could produce specific and stable fingerprints that could be used for phage genotyping.

## Results

### BOXA-PCR for phage fingerprinting

**Fig. 1:**
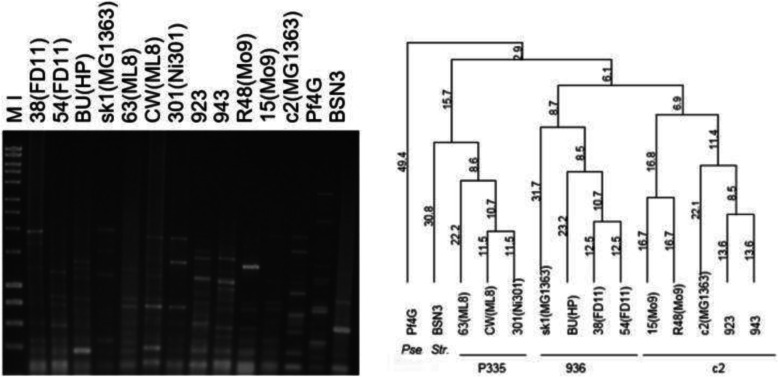
BOXA2R-PCR phage fingerprint profiles. *Pseudomonas* phage Pf4G; *Str. thermophilus* phage BSN3; *Lc. lactis* phages: 38 and 54 (host *Lc. lactis* ssp. *lactis* biovar diacetylactis phage FD11); 63 and CW (host *Lc. lactis* ssp. *lactis* ML8); 301 (host *Lc. lactis* ssp. *lactis* Ni301); BU (host *Lc. lactis* ssp. *cremoris* HP); 923 and 943 (*Lc. lactis* 112); 15 and R48 (host *Lc. lactis* ssp. *cremoris* Mo9). The reference phages were sk1 for the 936-group and c2 for the c2-group. The corresponding dendrogram was generated using the UPGMA method. Marker (M I) - HyperLadder I (Bioline)

The potential of repetitive-PCR as a fingerprinting method was initially tested with the BOXA2R primer on a set of *Lc. lactis* phages; one *Pseudomonas* phage and one *Str. thermophilus* phage (see Fig. 1). Lactococcal phages were selected to cover isolates from different sources and comprised representatives of the three main species (936, P335 and c2), while the phages that infect two other bacterial species were included to explore whether the method would be applicable to other phage types. The analysis showed that lactococcal phages of the same species clustered together. For example, phage 38 (host *Lc. lactis* ssp. diacetylactis FD11) and phage BU (host *Lc. lactis* ssp. *cremoris* HP) that both belong to the 936-species formed one group together with the reference 936-type phage sk1. Also, the P335-type phages, 63 and CW that infect *Lc. lactis* ssp. *lactis* ML8, and 301 that infects *Lc. lactis* ssp. *lactis* Ni301, clustered together. Within the c2-like and 936-like clusters, phages that infected the same host, such as 38 and 54 or 15 and R48 (host *Lc. lactis* ssp. *cremoris* Mo9) were more related, while within a P335 cluster, phages to the same host were not the most closely related. Out of the three lactococcal phage groups, *Str. thermophilus* phage BSN3 appeared most related to P335 phages, while *Pseudomonas* phage Pf4G was an outlier. Similar phage groupings were also generated using the BOXA1R primer, except that phage BU (HP) consistently generated a single intense fragment of ~ 300 bp, which affected the relationship analysis (results not shown).

### Comparison of the BOXA-PCR results with phage sequencing data

**Fig. 2:**
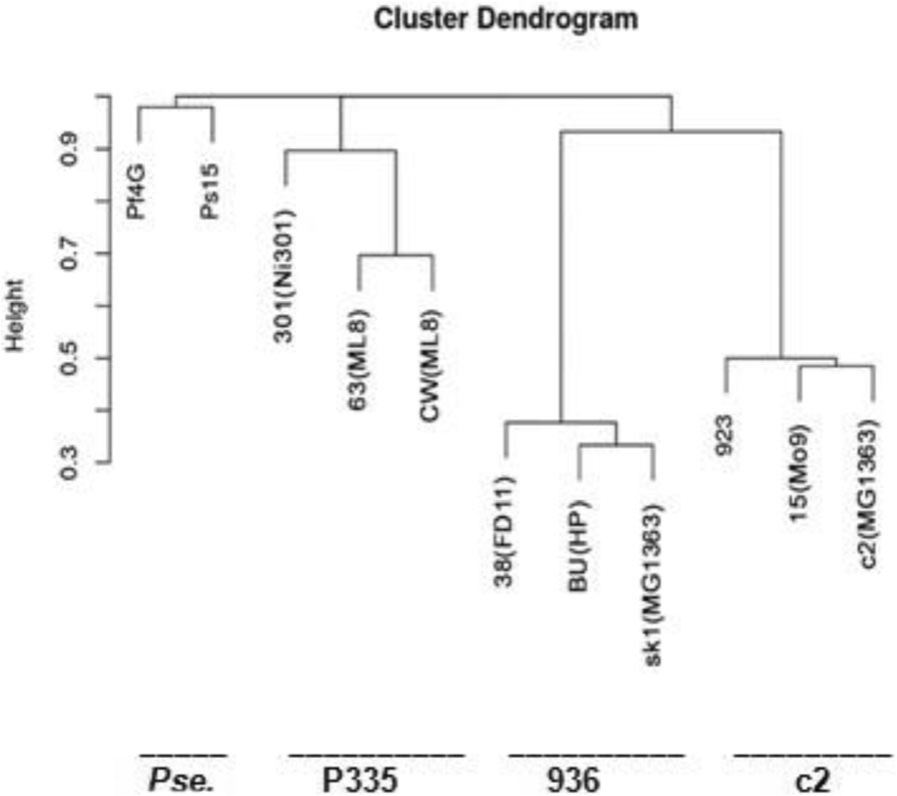
Hierarchical clustering of the predicted proteomes of the sequenced phages based on the presence/absence of pVOGs. The dendrogram was calculated using the complete linkage method

To determine whether phage relatedness inferred from BOXA-PCR fingerprinting could be supported by another classification method, the genomes of eight lactococcal (38(FD11), BU (HP), 301(Ni301), 63(ML8), CW (ML8), 15(Mo9), 923 and R48(Mo9)) and two *Pseudomonas* (Pf4G and Ps15) phages were sequenced (manuscript in preparation), and the sequences compared. The genome of R48 was not included into the final analysis due to difficulties with its assembly. The hierarchical clustering was performed using the Prokaryotic Virus Orthologous Groups (pVOGs, formerly termed Phage Orthologous Groups, POGs) resource [27]. The annotation of pVOGs was made using the predicted proteins and the hierarchical clustering was performed on these annotations. It resulted in clustering of lactococcal phages comparable to that seen with BOXA-PCR, where 936-, P335-, and c2-type phages formed well defined separate clusters. The *Pseudomonas* phages were placed outside these groups and were unrelated (see Fig. 2).

An alternative approach based on the correlation of tetranucleotide signature frequencies, that showed high discriminatory power comparing genome fragments even when alignments are not likely [28], produced the same correlations between the tested phages (see Fig. 1, Additional file 1).

### BOXA-PCR method validation

The method was investigated and validated on a group of well characterised *Lc. lactis* phages using both BOXA1R and BOXA2R primers (see Table 1, Additional File 2). The reproducibility of the method was explored using phage propagated at two different temperatures (30 °C and 37 °C) with the DNA being isolated by two protocols. The details of the statistical analysis descriptives performed using the non-parametric Mann-Whitney test are presented in the Supplementary material (see Additional file 3).

Repetitive-PCR with both BOXA1R and BOXA2R primers resulted in reproducible DNA fingerprint profiles of the tested phages, which also included lambda, phiX174 and T4 phages. The similarity index of the BOXA1R-PCR replicates ranged from 90.2–100% and of BOXA2R-PCR replicates from 89.4–100%. The difference in performance between these two primers was not statistically significant (*p* = 0.279) (see Table 1, Additional File 3).

#### Influence of the DNA isolation method on the phage profile

**Fig. 3:**
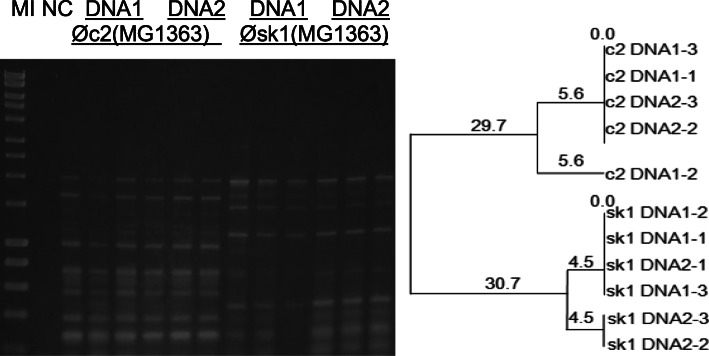
Reproducibility testing of the BOXA2R-PCR using the DNA of lactococcal phages c2 and sk1**.** Both phages were propagated on *Lc. lactis* ssp. *cremoris* MG1363 at 30 °C. The corresponding dendrogram was generated using the UPGMA method. Three replicates per each DNA isolated by the phenol-chloroform procedure (DNA1) and the Qiagen kit (DNA2) were tested. Marker (M I) - HyperLadder I (Bioline). NC- negative control

**Fig. 4:**
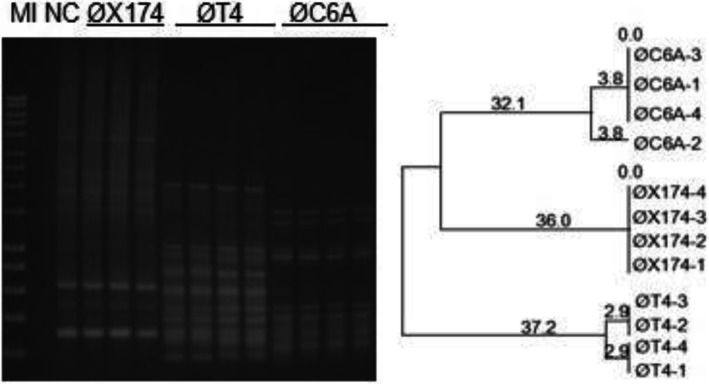
BOXA1R-PCR reproducibility testing. The templates used in PCR reactions included phiX174 RF1 DNA (Thermo Fisher), ØT4 lysate and DNA of ØC6A amplified on *Lc. lactis* ssp. *lactis* C6. The corresponding dendrogram was generated using the UPGMA method. Marker (M I) - HyperLadder I (Bioline). NC- negative control

**Fig. 5:**
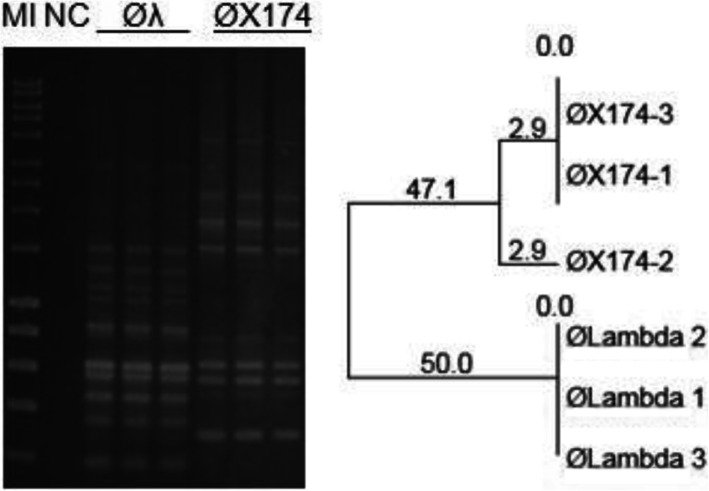
Reproducibility testing of the BOXA1R-PCR using commercially isolated DNA. The reactions were performed using 30 ng DNA of the ØLambda (Øλ) (Thermo Fisher) and 25 ng of ØX174 (phiX174, Thermo Fisher) per reaction. The corresponding dendrogram was generated using the UPGMA method. Marker (M I) - HyperLadder I (Bioline). NC- negative control

Separate PCR amplifications from the same DNA source generated reproducible fingerprints with each BOXA2R primer (see Fig. 3) and BOXA1R (see Fig. 4). The replicates showed very similar (see Fig. 3, Øsk1 and Fig. 4, ØT4) or indistinguishable (see Fig. 4, ØC6A and Fig. 5, ØLambda) profiles. No statistically significant difference (*p* = 0.08) was observed in the fingerprint profiles of the same phage using the two DNA preparation methods, phenol-chloroform and Qiagen commercial kit (see Table 3, Additional File 3).

Rep-PCR produced stable profiles when the same phage DNA was amplified at different times on separate gels, although band migration on gels may fluctuate. An example, ØX174 amplified with BOXA1R is presented in Figs. 4 and 5. The two gels appeared different due to different concentrations of DNA used in the PCR reactions, 100 ng (Fig. 4) vs 25 ng (Fig. 5). When the migrating bands are compared against the marker it can be seen that the banding patterns are consistent between the two gels.

#### Influence of the propagation temperature on the phage profile

Minor variations in the fingerprint patterns of the same phage cultivated and amplified at two temperatures, 30 °C and 37 °C, were observed, however the differences were not significant (*p* = 0.527) (see Table 2, Additional File 3). The variability in band intensity, which was occasionally seen, was not associated with the incubation temperature as there was no clear separation between the 30 °C or 37 °C clusters (see Fig. A in Additional File 4).

#### Influence of the propagating host on the phage profile

**Fig. 6:**
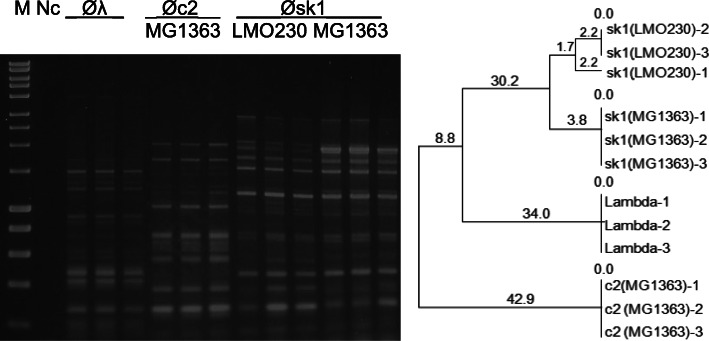
Reproducibility (*n* = 3) testing of the BOXA2R-PCR. Øc2 was propagated on *Lc. lactis* ssp. *cremoris* Mg1363 and Øsk1 was propagated on the *Lc. lactis* ssp. *cremoris* LMO230 and MG1363 at 30 °C. PCR amplifications were performed using DNA1. Three replicates of the phage Lambda were generated with 12 ng of DNA (Thermo Fisher). The corresponding dendrogram was generated using the UPGMA method. Marker (M I) - HyperLadder I (Bioline). NC- negative control

The host on which a phage was amplified slightly altered the phage profile, such as seen in the BOXA2R-PCR profiles of the Øsk1 amplified on two bacterial hosts: *Lc. lactis* ssp. *cremoris* LMO230 and MG1363 (see Fig. 6) or the ØP087 amplified on *Lc. lactis* ssp. *lactis* strains ML8 and C10 (see Fig. B in Additional File 4). Slightly differing bands were seen in the ~ 2 Kbp region, where Øsk1(MG1363) displayed a single, pronounced band whereas Øsk1(LMO230) displayed two smaller bands instead (96% similarity). The differences in the ØP087 profiles were detected in the ~ 800–1000 bp range, which resulted in the 90% similarity between amplifications when propagated on the two hosts.

Similarly, one band difference between the profiles of the same phage propagated on two different hosts was also observed with BOXA1R. For example, Øc6A amplified on the *Lc. lactis* ssp. *lactis* C6 and C10 differed by the presence of a ~ 580 bp band, which was visible only for the C6 host (results not shown).

### Application of BOXA-PCR genotyping for screening phage isolates

**Fig. 7:**
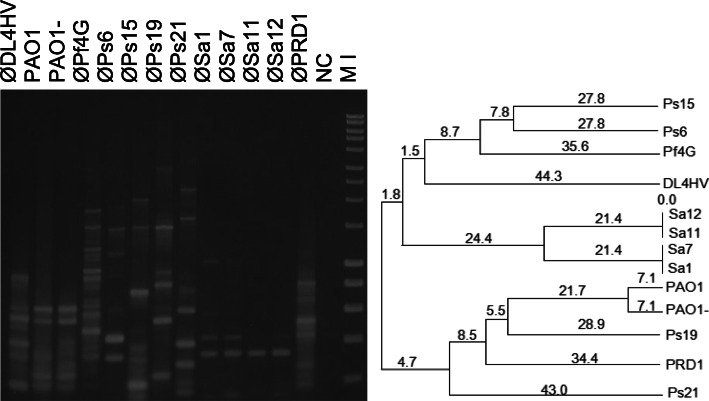
BOXA1R-PCR fingerprint profiles generated using different template sources. The purified DNA of the *Halorubrum* phage DL4HV and *Ps. aeruginosa* Pf4G; lysates of the *Ps. aeruginosa* phages Ps6, Ps15, Ps19 and Ps21; *Staph. aureus* phages Sa1, Sa7, Sa11 and Sa12; and a plaque of the enteric phage PRD1 were used. BOXA1R-PCR profiles of the two bacterial strains were also included: *Ps. aeruginosa* PAO1 (wild type) and PAO1- (prophage-free mutant). The corresponding dendrogram was generated using the UPGMA method. Marker (M I) - HyperLadder I (Bioline). NC- negative control

**Fig. 8:**
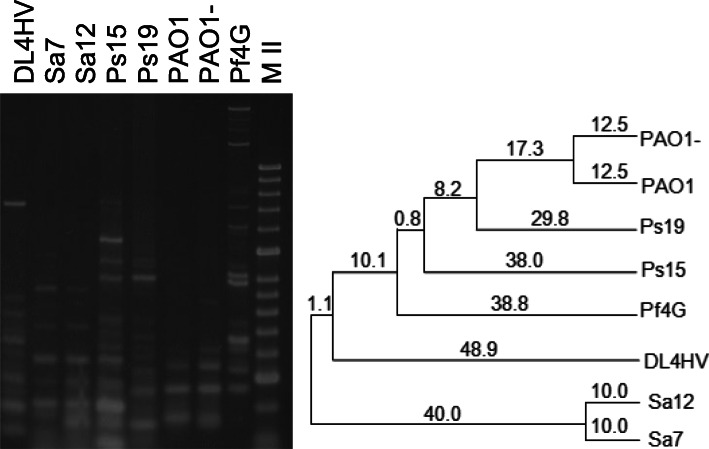
BOXA2R-PCR fingerprint profiles generated using different template sources. The purified DNAs of the *Halorubrum* phage DL4HV and *Ps. aeruginosa* phage Pf4G; lysates of the *Ps. aeruginosa* phages Ps15 and Ps19; and *Staph. aureus* phages Sa7 and Sa12 were used. Two bacterial strains were also included: *Ps. aeruginosa* PAO1 (wild type) and PAO1- (prophage-free mutant). The corresponding dendrogram was based on the UPGMA method. Marker (M II) - HyperLadder II (Bioline). NC- negative control

Repetitive-PCR using either BOXA1R or BOXA2R repetitive primer was further tested on a number of ecological isolates with distinctive fingerprint profiles being generated for all tested phage (see Figs. 7–8).

The amplification of *Staphylococcus aureus* phages with BOXA1R primer produced the lowest number (2–5) of PCR fragments (see Fig. 7). All four isolates appeared highly related, with there being no difference in profiles for Sa1 and Sa7, and for Sa11 and Sa12, whereas the similarity between these two pairs was 90%. Contrary to the *Staph. aureus* phages, the four *Pseudomonas* phages (Ps6, Ps15, Ps19 and Ps21) produced four unique fingerprint profiles. The Ps6, Ps15, Ps19 phages all exhibited one band of ~ 1.5 kb in common, whereas all other bands were exclusive to each phage isolate.

The PCR profiles of DNA extracted from the purified particles of the lytic ØPf4G obtained using the two boxA primers were compared to that of the wild-type *Ps. aeruginosa* PAO1, and the mutant of PAO1 created by the removal of the complete filamentous Pf4 prophage genome (PAO1-) [31]. While the band patterns of both bacterial strains were similar with either boxA primer, the phage Pf4G displayed a very different profile to these bacteria and yielded DNA fragments of substantially different size and length (see Figs. 7 and 8). This indicated that the superinfective ØPf4G may have undergone significant DNA rearrangement compared to its prophage/temperate form.

Successful amplification of the purified DNA of an archeal phage, DL4HV, which infects *Halorubrum* sp. and some other halobacteriaceae, was also achieved using the BOXA- PCR method (see Figs. 7 and 8).

### Sequencing of the BOXA-PCR fragments

Eight PCR products randomly selected from fingerprints of different phage isolates were sequenced with either BOXA1R or BOXA2R primer (see Table 1, Additional File 5) to determine the sequence of the genes contained in the rep-PCR products. This experiment resulted in the identification of encoded proteins that are clearly linked to the phages they originated from, although with varying levels of homology (37–89%). The lower levels of homology may have resulted from technical issues or from alternative binding sites of the BOXA primers to the rep-PCR products, thereby producing overlapping sequences that are difficult to deconvolute.

To further validate the presence of the identified genes in the phage genomes, a series of primers were designed to target the exact coding sequences obtained by the BLAST analyses (see Table 2, Additional File 5). The second round of amplifications of four fragments with gene-specific primers generated PCR products that matched the size of the targeted genes (see Fig. 1, Additional File 5). Sequencing of these products confirmed the genes identified in the BLAST results (see Figs. 1–4, Additional File 6).

The comparisons of results obtained by sequencing selected fragments using both the boxA repetitive primers and primers specifically designed for particular annotated genes are presented in Table 1.

**Table 1:** BOXA-PCR fragment sequencing and confirmatory results

FRAGMENT NAME (Primer)	Rep-PCR fragment size-gel assessment (bp)	UniProt KB annotation of rep-PCR fragment (Top BLAST result)	Rep-PCR BLAST results	Confirmatory BLAST results using primers designed to amplify the putative CDS*
ØX174-1 (BOXA1R)	650	P03631 **Replication-associated protein A (**Enterobacteria phage phiX174)^a^	E-value: 1.9e-91 Score: 745 Ident.: 89.1%	E-value: 0.0 Score: 1819 Ident.: 93.30%
ØT4-2 (BOXA2R)	700	A0A376YLU8 Putative baseplate structural protein (Escherichia coli)	E-value: 1.7e-13 Score: 188 Ident.: 36.7%	E-value: 3.9e-133 Score: 1004 Ident.: 98.50%
ØLambda-1 (BOXA2R)	450	P03689 **Replication protein P** (Escherichia phage lambda)^a^	E-value: 1e-15 Score: 166 Ident.: 43.2%	E-value: 3.6e-156 Score: 1133 Ident.: 100.00%
Øc2-1 (BOXA1R)	1200	Q38305 **Probable tape measure protein (**Lactococcus phage c2)	E-value: 5.8e-17 Score: 145 Ident.: 66.7%	E-value: 0.0 Score: 1529 Ident.: 94.70%

*refer to Tables 1 and 2 (Additional file 5) for details

## Discussion

This work has demonstrated that the BOXA-PCR method is applicable for phage fingerprinting and is discriminatory and reproducible under various conditions. The discriminatory capacity of the method was corroborated against the whole genome sequencing data of selected *Lc. lactis* and *Pseudomonas* phages. Phage relationships deduced from each method compared well and resulted in clustering of lactococcal phages according to their species while *Pseudomonas* phages were clearly identified as a separate group.

Reproducibility of the BOXA-PCR was demonstrated using both BOXA1R and BOXA2R primers on a set of selected known phages amplified on different hosts and/or amplified in the same host after cultivation at different temperatures (30 °C and 37 °C). Consistent fingerprint profiles were obtained from phage DNA purified by two protocols (*p* = 0.08). The amplification of a phage on different hosts resulted in some small, but obvious differences. These variations likely reflect the small genetic differences between the host strains, such as in the case of *Lc. lactis* ssp. *cremoris* MG1363 and LMO230, the plasmid-free and prophage-cured derivatives of their closely related parent strains NCDO712 and C2 [32]. This aspect may represent a limitation of the method and requires further work, including investigating fingerprints of phages amenable to propagation on both genetically similar and dissimilar strains.

BOXA-PCR was tested on a range of phages that infect a variety of bacteria, for example *E. coli*, *Pseudomonas* sp., *Staphylococcus* sp., *Lc. lactis*; and also the haloarchaea algae *Halorubrum.* Both the BOXA1R and BOXA2R primers used in the PCR amplification of phage DNA produced distinctive individual fingerprint profiles for all tested isolates. This broad application may be due to the lower GC content of these primers and the associated optimal annealing temperature of 40 °C [7]. This decreased stringency may have enabled the amplification of various phage types. It is suggested that potentially other repetitive primers, for example ERIC primers for enterococcal phages, could be more suitable for the genotyping of other bacteriophage types.

The method also appears useful for application to phages that are difficult to characterize by other genetic methods. For example, the DNA of the CR1 phage has previously been shown to be refractory to both restriction endonuclease digestion and to genome sequencing, which has prevented its genomic characterization [33]. Fingerprinting analysis with BOXA1R showed that it was unrelated to the *E. coli* ØT4 phage (results not shown). *Lc. lactis* ssp. *lactis* Ø15(Mo9) and some other lactococcal phages investigated in this study were also resistant to cutting by several restriction enzymes hence the direct determination of genetic relationship between various lactococcal phages by restriction enzyme digestion method was not possible.

Phage template DNA obtained from different sources (such as purified phage DNA, phage lysates or a single picked plaque from an agar plate) generated comparable BOXA-PCR products. These findings suggest the method would have utility and convenience for routine phage typing. However, when unpurified phage preparations are used as templates, such as lysate that has not been pre-treated with DNase or a plaque directly picked from the agar plate, there is a possibility that traces of host DNA may be co-amplified and contaminate phage fingerprint profile. The incorporation into the method of appropriate controls to account for any host DNA contamination could provide a solution to this limitation.

The sequencing of PCR fragments produced with the boxA primers verified the phage origin of the amplified DNA. The majority of the genes identified during the BLAST analyses against the UniProt KB database [34] encoded phage-associated proteins involved in different viral functions, such as the Replication protein A in ØX174 and the Replication protein P in ØLambda, respectively, or baseplate proteins (ØT4 and Ø301). Phage-originating genes identified from the nucleotide sequences might facilitate future work to identify and locate the repeats and elucidate the potential repetitive primer binding sites on the target genomes.

In terms of speed and simplicity the repetitive-PCR described in this work is comparable to RAPD-PCR. Both methods allow for a whole genome comparison. Rep-PCR is commonly applied to bacteria but has been reported as a complement to RAPD for phage characterization [35]. Although RAPD-PCR method has been used to examine genetic diversity of various bacterial and algal phages [18, 21, 22, 35, 36] it has been associated with poor reproducibility [37] and hence requires extensive optimization [21, 37]. This involves selection of a suitable decamer primer [22, 35] followed by optimization of PCR conditions, such as primer and MgCl_2_ concentration, annealing temperature and standardization of template concentrations [22, 38]. Furthermore, to increase the sensitivity of the RAPD assay, the pooling of patterns from at least two amplifications with different random primers has often been applied [35, 38]. RAPD cannot discriminate closely related phages and the amplification of the phage and host DNA has not been possible under the same RAPD-PCR assay conditions [22]. Our findings demonstrated that the use of BOXA-PCR under defined conditions could achieve better discrimination, higher reproducibility with fewer optimisation steps and would be more broadly applicable than RAPD-PCR for use in phage genotyping.

## Conclusion

The findings of this study suggest that repetitive-PCR could be a useful high resolution method for bacteriophage genotyping. Repetitive-PCR, preferably using specific primers to BOX sequences: BOXA1R and BOXA2R, is applicable to the genetic identification of an isolated phage and for the investigation of genetic relationships between phage genotypes. The technique could well be used in a phage management program in addition to multiplex PCR for phage species determination where the knowledge of genetic profiles of circulating and emerging phages could be advantageous for the selection of the most appropriate strategies to address contamination problems. It could also be used to complement other phage genotyping methods, such as RFLP, and would be particularly useful for analysing phage which appear refractory to digestion with restriction endonucleases. Given the abundance of phage in the environment [20], having a rapid, inexpensive method for genetic characterization of both phage and its bacterial hosts could be useful especially when preliminary genotyping large numbers of phage isolates is required for screening purposes.

## Methods

### Bacteria

*Lc. lactis* ssp. *lactis* Ni301 and *Lc. lactis* ssp. *cremoris* Mo9 were isolated from dairy products. *Lc. lactis* ssp. *lactis* biovar diacetylactis FD11 was isolated from the mesophilic starter type culture Flora Danica (FD). *Lc.lactis* ssp. *cremoris* HP and UK712, and *Lc. lactis* ssp. *lactis* biovar diacetylactis ML8 were retrieved from our internal culture collection. *Lc. lactis* ssp. *lactis* 112, C6, C10 and IL1407 were kindly provided by Dr. Jasna Rakonjac, Massey University, NZ. *Pseudomonas aeruginosa* PAO1 (wild type) and PAO1- (free of endogenous prophage Pf4) were kindly provided on plates by Dr. Vanessa Huron, The Centre for Marine Bio-Innovation and the School of Biotechnology and Biomolecular Sciences (BABS).

The strains were grown anaerobically for 24–48 h at 30 °C on M17 agar (Oxoid) or M17 broth supplemented with 0.5% lactose (LM17) [39]. Stock cultures were prepared in 9.5% (w/v) autoclaved (121 °C for 15 min) reconstituted skim milk and kept at − 80 °C.

### Bacteriophages

The *Lc. lactis* phages used in the study were isolated from dairy whey samples (see Table 2) with other phage types provided by external sources (see Table 3). Phage preparations provided by external sources were analysed by PCR in the form they were supplied without any further modification. The phage coding system for the phages isolated in this study was based on designating the phage name followed by the name of the bacterial strain on which that phage was propagated (in brackets).

**Table 2:** Bacteriophage samples isolated from whey samples

Phage code	Host Species	Titer (pfu/ml)	Year of isolation	Phage species	Phage plaque description	Source
15(Mo9)	*Lactococcus lactis* ssp *cremoris* Mo9	2.00E+08	2016	c2+P335^*^	2 mm clear + 1 mm zone	isolated in this study
R48(Mo9)	*Lc. lactis* ssp *cremoris* Mo9	1.90E+08	2016	c2	2 mm and 4 mm clear	isolated in this study
301(Ni301)	*Lc. lactis* ssp *lactis* Ni301	2.20E+10	2016	P335	1 mm clear	isolated in this study
54(FD11)	*Lc. lactis* ssp *lactis* var diacetylactis FD11	2.80E+09	2016	936	3 mm clear + 2 mm margin	isolated in this study
38(FD11)	*Lc. lactis* ssp *lactis* var diacetylactis FD11	6.00E+09	2016	936	2 mm clear + 2 mm margin	isolated in this study
BU(HP)	*Lc. lactis* ssp *cremoris* HP	7.20E+09	2016	936	6 mm clear	isolated in this study
63(ML8)	*Lc. lactis* ssp *lactis* var diacetylactis ML8	3.70E+09	1981	P335	2 mm clear, sharp edge	BABS, UNSW culture collection
CW(ML8)	*Lc. lactis* ssp *lactis* var diacetylactis ML8	1.50E+08	1981	P335	1mm clear	BABS, UNSW culture collection
BSN3 (France)	*Streptococcus thermophilus* BSN3	5.30E+08	1988	-	-	BABS, UNSW culture collection

*15(Mo9) has been identified as a *Lactococcus* virus *unclassified c2-like* (unclassified *Ceduovirus*) bacteriophage by DNA sequencing (manuscript in preparation)

- Not determined

**Table 3:** Bacteriophage samples provided by external sources

Phage code	Host Species	Preparation	Provider
Pf4G	*Pseudomonas aeruginosa*	purified DNA	A/Prof. Scott Rice, UNSW
DL4HV	*Halorubrum* sp.	purified DNA	Dr Susanne Erdmanne, Max Planck Institute for Marine Microbiology, Germany
T4	*Escherichia coli*	phage lysate	Dr Nicola Petty, The iThree institute, UTS
CR1	*Citrobacter rodentium*	phage lysate	Dr Nicola Petty, The iThree institute, UTS
Ps6, Ps15, Ps19, Ps21	*Pseudomonas* sp.	phage lysate	Dr Lisa Elliott, AusPhage
Sa1, Sa7, Sa11, Sa12	*Staphylococcus aureus*	phage lysate	Dr Lisa Elliott, AusPhage
923, 943	*Lactococcus lactis*	phage lysate	A/Prof. Jasna Rakonjac, Massey Univ., NZ
PRD1	*E. coli*	plate	Culture collection, BABS, UNSW

### Phage propagation, amplification, and titering

The lysates of the isolated phages were prepared from single plaques picked from sensitive host lawns of double layer LM17 agar plates containing 10 mM CaCl_2_ [39] and were purified twice. Following the amplification and filtration with a 0.22 μm sterile Millipore filter, broth lysates with titers equal or higher than 10^8^ pfu/ml were stored in 1.5 ml cryovials at − 20 °C.

### Phage purification and DNA isolation

Crude phage lysate (1.5 ml) was treated with 10 μg/ml RNase A and 1 μg/ml DNase I (Sigma) final concentration for 30 min at 37 °C to remove bacterial nucleic acids. Following centrifugation at 22000 X G for 10 min, the phage particles in the supernatant were PEG-salt precipitated and further purified as previously described [40].

### Multiplex PCR for phage typing

The determination of lactococcal phage type was performed by multiplex PCR as described by Labrie and Moineau (2000) [16] using isolated phage DNA as template.

### Repetitive polymerase chain reaction (rep-PCR)

Phage genotyping was performed using puReTaq Ready-To-Go Polymerase Chain Reaction (PCR) Beads (GE Healthcare) with either BOXA1R (5′-CTACGGCAAGGCGACGCTGACG-3′) or BOXA2R (5′-ACGTGGTTTGAAGAGATTTTCG-3′) [7] as a single primer.

For each reaction, 50 pmol of the single primer BOXA1R or BOXA2R, 50–100 ng *Lc. lactis* phage DNA in DNase/RNase - free water at a final volume of 25 μl was added to a tube containing a single PCR bead. The rep-PCR method was also tested using phage lysates (10^5^ pfu/ml) and plaques as DNA sources. The negative control reaction contained primer only, with water substituted for the template DNA. PCR amplifications were performed in an automated thermal cycler (Eppendorf) with an optimal cycling program set for each primer (see Table 4). The amplification products (5 μl) were electrophoresed on 15 × 20 cm 1.2% (w/v) molecular grade agarose (Bio-Rad) gels in 1 x TAE (Tris-acetate, EDTA, pH 8.1) at a constant 100 V for 3 h. The amplicons were assessed against the molecular size marker HyperLadder I (M I) or HyperLadder I (M II) (Bioline). After staining with GelRed 3 x staining solution in water (Biotium) for 15 min, the gels were photographed with a camera attached to a tripod mounted on a UV-transilluminator (Ultra Lum).

**Table 4:** PCR amplification conditions for the primers, BOXA1R and BOXA2R

Primer name	Amplification conditions	Reference
BOXA1R	2 min at 92 °C, 35 cycles of: 30 s at 92 °C, 1 min at 40 °C, 2 min at 72 °C; 5 min at 72 °C	Zavaglia et al., 2000 [29]
BOXA2R	7 min at 95 °C, 35 cycles of: 30 s at 90 °C, 1 min at 40 °C, 8 min at 65 °C; 16 min at 65 °C	Malathum et al., 1998 [30]

#### Method validation

Reproducibility of the rep-PCR method using BOXA1R and BOXA2R was tested on a set of known phages under defined conditions (see Table 1, Additional File 2). *Lc. lactis* ssp. *lactis* Ø301, which was isolated in this work was sequenced and included in the reproducibility study. Phage DNA was isolated following two protocols: the protocol described above (referred to as DNA1) and QIAamp DNA Blood Mini Kit (Qiagen) (referred to as DNA2). For the preparation of DNA2, phage lysate was pre-treated with DNase I and concentrated with PEG-salt solution before the Qiagen DNA viral DNA purification protocol was followed. At least three separate replicates from the same DNA template for each strain were used for PCR amplifications.

### Statistical analysis

Gel images of the BOXA1R- and BOXA2R-PCR fingerprints were analyzed with the PyElph 1.4 software [41]. The generated dendrograms were based on the Unweighted Pair Group Method with Arithmetic Mean (UPGMA) cluster analysis applied on the computed distance matrix [41]. Due to the log-normal data distribution, the statistical analysis was performed using the non-parametric Mann-Whitney test in IBM SPSS Statistics Version 25. The effects of the two DNA templates (DNA1 and DNA2) and two phage growth temperatures (30 °C vs 37 °C) on the reproducibility of the fingerprint profiles were tested at *p* < 0.05.

#### Phage genome sequencing and bioinformatic analysis

The sequencing of phage DNA was performed in the Ramaciotti Center for Genomics, UNSW, Sydney on a MiSeq system (Illumina) (1x MiSeq reagent kit v2, 2 × 150 bp Nano Sequencing Run). The sequencing library was prepared with 10x Nextera XT DNA Library preparation kit (Illumina). Phage genomes were assembled with SPAdes v3.13.1 [42]. Gene-calling was performed with multiPhATE v1.0 [43]. Predicted proteins were annotated against Prokaryotic Virus Orthologous Groups (pVOGs, downloaded Nov. 2019) [27] with hmmscan (HMMER v3.2.1) [44].

Cluster analysis on the presence-absence of pVOGs was performed in R v3.6.1 [45] with hclust on a distance matrix calculated with the “binary” method in the stats package.

Correlation based on tetranucleotide composition was calculated with pyani v0.2.9 [46].

### Sequencing of the PCR bands

Randomly selected individual DNA amplicons (see Table 1, Additional File 5) generated with either primer were separated from a mixture of other PCR products by applying the band stab method [47]. The subsequent re-amplification with the same primer in a new PCR reaction was run on a gel to verify that a single band of the same size was obtained. The size of the amplified fragment was measured against the molecular size marker, and the concentration and purity were determined by Nanodrop. After purification with GenElute PCR Clean-Up kit (Sigma), Sanger sequencing of the selected amplicons was performed in the Ramaciotti Centre for Genomics using either BOXA1R or BOXA2R as a sequencing primer. BLAST runs of the obtained nucleotide sequences against the UniProt KB database resources were used to deduce the protein sequences and their functional information [34].

### Validation of the phage origin of rep-PCR products

Primers targeting specific phage genes were designed based on the results from BLAST analyses (see Table 2, Additional File 5). PCR was performed using the puReTaq Ready-To-Go Polymerase Chain Reaction (PCR) Beads (GE Healthcare) and the appropriate purified phage DNA as template. The sizes of PCR products were evaluated by electrophoresis in 1% (w/v) agarose gels against the molecular size marker. Selected PCR products were sequenced in the Ramaciotti Centre for Genomics for final validation using the corresponding forward primer as the sequencing primer. Sequencing results were aligned against the coding sequence of genes identified during BLAST analyses using the MAFFT algorithm in the Benchling platform [48].

## Supplementary information

**Additional file 1 Analysis of the sequenced phage genomes based on tetranucleotide frequencies.** This file provides the figure that shows a Pearson correlation of tetranucleotide frequencies including the clustering trees and heatmaps with Pearson correlation values.

**Additional file 2 List of phages used in the reproducibility study.** This data outlines the phages used in the study, which strain they were propagated on, and the culture conditions.

**Additional file 3 Statistical analysis of the BOXA-PCR for phage fingerprinting.** This file provides the details of the statistical analysis of the reproducibility testing.

**Additional file 4 Influence of the propagating temperature and propagating host on the phage profile.** This file provides the BOXA2R-PCRfingerprint profiles of lactococcal phages 712 and P087, which show the influence of the propagating temperature and their hosts on pattern reproducibility.

**Additional file 5 Sequenced phage fragments.** This file provides the list of sequenced phage fragments and details relating to their analysis, including; the primers used for the amplification of selected phage genes; results from the second round of PCR amplifications and BLAST analyses that used gene-specific primers; and a figure showing PCR amplifications of the selected phage DNA fragments using the forward and reverse primer pair corresponding to the annotated genes in each phage.

**Additional file 6 Sanger sequencing of the BOXA-PCR fragments.** This file provides the alignment of the sequencing of the PCR products to the sequences of the corresponding proteins.

## Data Availability

All data generated or analysed during this study are included in the Results section of this published article and its supplementary information files (Additional files 1–6).

## Abbreviations

Rep-PCR: Repetitive-PCR
MLST: Multilocus sequence typing
RFLP: Restriction fragment length polymorphism
DGGE: Denaturing gradient gel electrophoresis
RAPD-PCR: Random amplification polymorphic DNA
ERIC: Enterobacterial repetitive intergenic consensus
UPGMA: Unweighted pair group method with arithmetic mean
pVOGs: Prokaryotic Virus Orthologous Groups
