# Supplementary material for: Bacteriophage genotyping using BOXA repetitive-PCR
Source: BMC Microbiol. 2020 Jun 11;20:154. doi: 10.1186/s12866-020-01770-2 (PMC7291552; doi:10.1186/s12866-020-01770-2)
Supplement: Supplementary file 1 — Additional file 1 Analysis of the sequenced phage genomes based on tetranucleotide frequencies. This file provides the figure that shows a Pearson correlation of tetranucleotide frequencies including the clustering trees and heatmaps with Pearson correlation values. [file 12866_2020_1770_MOESM1_ESM.pdf]

Additional file 1.

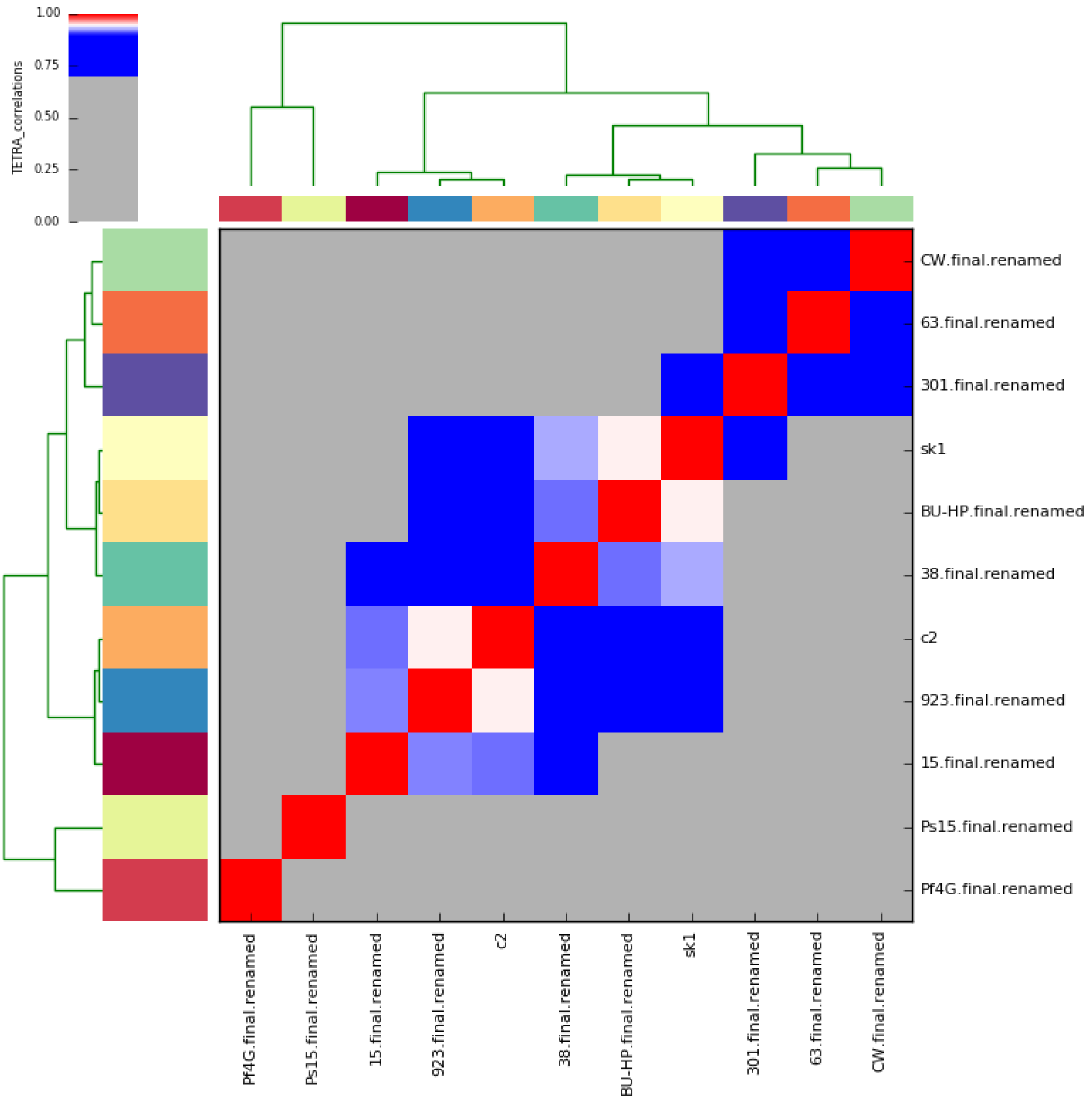

Figure 1. Pearson correlation of tetranucleotide frequencies of the sequenced phages. Trees show clustering based on the tetranucleotide frequencies. Heatmaps show Pearson correlation values.
