# Supplementary material for: Bacteriophage genotyping using BOXA repetitive-PCR
Source: BMC Microbiol. 2020 Jun 11;20:154. doi: 10.1186/s12866-020-01770-2 (PMC7291552; doi:10.1186/s12866-020-01770-2)
Supplement: Supplementary file 2 — Additional file 2 List of phages used in the reproducibility study. This data outlines the phages used in the study, which strain they were propagated on, and the culture conditions. [file 12866_2020_1770_MOESM2_ESM.pdf]

Additional file 2.

Table 1. List of phages used in the reproducibility study

**Phage name**	**Propagated on the strain**	**Conditions**
Øc2[a]	*Lc.lactis* ssp *cremoris* Mg1363	DNA1and DNA 2 at 30°C
Øc2	*Lc.lactis* ssp *cremoris* Mg1363	DNA1 at 37°C
Øc2	*Lc.lactis* ssp *cremoris* C2	DNA1 and DNA2 at 30°C
Øc2	*Lc.lactis* ssp *cremoris* C2	DNA1 and DNA2 at 37°C
Øsk1	*Lc.lactis* ssp *cremoris* Mg1363	DNA1 and DNA2 at 30°C
Øsk1	*Lc.lactis* ssp *cremoris* Mg1363	DNA2 at 37°C
Øsk1[b]	*Lc.lactis* ssp *cremoris* LMO230	DNA1 and DNA2 at 30°C
ØP087	*Lc.lactis* ssp *lactis* ML8	DNA2 at 30°C
ØP087[c]	*Lc.lactis* ssp *lactis* C10	DNA2 at 30°C
Øc6A[a]	*Lc.lactis* ssp *lactis* C6	DNA1 and DNA2 at 30°C
Øc6A	*Lc.lactis* ssp *lactis* C10	DNA1 and DNA2 at 30°C
Ø712	*Lc.lactis* ssp *cremoris* C2	DNA1 at 30°C
Ø712	*Lc.lactis* ssp *cremoris* C2	DNA1 at 37°C
Ø712[b]	*Lc.lactis* ssp *cremoris* NCDO712	DNA2 at 30°C
Ø712	*Lc.lactis* ssp *cremoris* NCDO712	DNA2 at 37°C
ØbIL67[a]	*Lc.lactis* ssp *lactis* IL1407	DNA1 and DNA2 at 30°C
ØT4[d]	-	lysate
ØX174[e]	-	DNA
ØLambda[e]	-	DNA
Ø301[f]	*Lc.lactis* ssp *lactis* Ni301	DNA1 and DNA2 at 30°C

DNA1 refers to the phage DNA isolated by the phenol/chloroform protocol

DNA 2 refers to the phage DNA isolated using the QIAamp DNA Blood Mini Kit (Qiagen)

a – Øc2 (host MG1363), ØbIL67(host IL1407), Øc6A(host C6) were provided by Dr Jasna Rakonjac, Massey University, New Zealand.

b- Øsk1(host LMO230) and Ø712(host UK712) were sourced from our internal UNSW culture collection.

c- ØP087 was purchased from the Félix d'Hérelle Reference Center for bacterial viruses of the Université Laval, Canada.

d- *E. coli* T4 phage lysate was obtained from Dr Nicola Petty, The iThree institute, University of Technology, Sydney.

e- PhiX174 RF1 DNA (0.5 µg/µl) and Lambda phage DNA (0.3 µg/µl) were purchased from Thermo Fisher Scientific, Australia.

f- Ø301(host Ni301) was isolated in the course of this study and was sequenced in the Ramaciotti Centre for Genomics, UNSW, Sydney.
