# Supplementary material for: Bacteriophage genotyping using BOXA repetitive-PCR
Source: BMC Microbiol. 2020 Jun 11;20:154. doi: 10.1186/s12866-020-01770-2 (PMC7291552; doi:10.1186/s12866-020-01770-2)
Supplement: Supplementary file 3 — Additional file 3 Statistical analysis of the BOXA-PCR for phage fingerprinting. This file provides the details of the statistical analysis of the reproducibility testing. [file 12866_2020_1770_MOESM3_ESM.pdf]

**Additional file 3.**

**Statistical analysis of the BOXA-PCR for phage fingerprinting**

**BOXA1R vs BOXA2R primer**

Table 1. SPSS output of the reproducibility analysis showing that there was no statistically significant difference between the replicates generated using BOXA1R vs BOXA2R primer (p=0.279).

**Descriptive Statistics**

| | N | Mean | Std. Deviation | Minimum | Maximum |
|---|---|---|---|---|---|
| Values | 117 | 98.2128 | 2.93565 | 89.40 | 100.00 |
| Tests | 117 | 1.5470 | .49993 | 1.00 | 2.00 |

**Mann-Whitney Test**

**Ranks**

| | Tests | N | Mean Rank | Sum of Ranks |
|---|---|---|---|---|
| Values | BOXA1R | 53 | 62.11 | 3292.00 |
| | BOXA2R | 64 | 56.42 | 3611.00 |
| | Total | 117 | | |

**Test Statistics**

| | Values |
|---|---|
| Mann-Whitney U | 1531.000 |
| Wilcoxon W | 3611.000 |
| Z | -1.086 |
| Asymp. Sig. (2-tailed) | .277 |
| Exact Sig. (2-tailed) | .279 |
| Exact Sig. (1-tailed) | .140 |
| Point Probability | .001 |

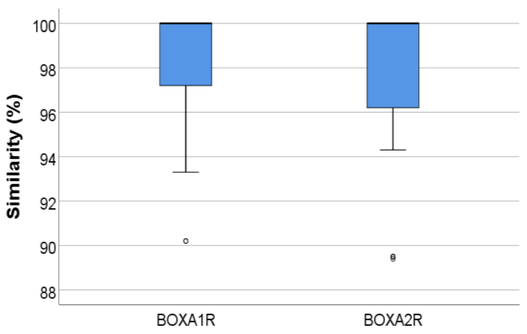


Figure 1. The plot illustrates 95% Confidence Intervals (CI) of dispersion estimates when using BOXA1R and BOXA2R primer

**DNA1 30°C vs DNA1 37°C**

Table 2. SPSS output of the reproducibility analysis of phage replicates where phage was amplified at 30°C and 37°C showing no significant difference between replicates ($p = 0.527$)

**Descriptive Statistics**

| | N | Mean | Std. Deviation | Minimum | Maximum |
|---|---|---|---|---|---|
| Data | 30 | 97.9233 | 2.87326 | 89.40 | 100.00 |
| Vars | 30 | 1.5000 | .50855 | 1.00 | 2.00 |

**Mann-Whitney Test**

**Ranks**

| | Vars | N | Mean Rank | Sum of Ranks |
|---|---|---|---|---|
| Data | 30C | 15 | 14.53 | 218.00 |
| | 37C | 15 | 16.47 | 247.00 |
| | Total | 30 | | |

**Test Statistics**

| | Values |
|---|---|
| Mann-Whitney U | 98.000 |
| Wilcoxon W | 218.000 |
| Z | -.681 |
| Asymp. Sig. (2-tailed) | .496 |
| Exact Sig. (2-tailed) | .567[a] |
| Exact Sig. (1-tailed) | .527 |
| Point Probability | .264 |

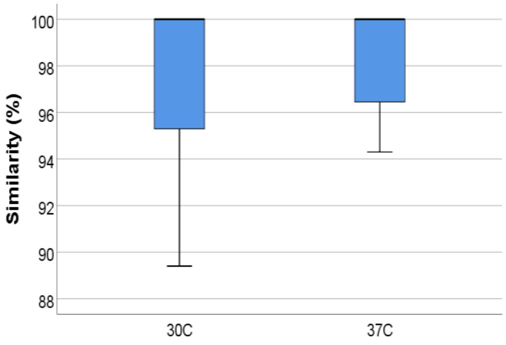


[a]. Not corrected for ties.

Figure 2. The plot illustrates 95% Confidence Intervals (CI) of dispersion estimates when using phage amplified at 30°C and 37°C as templates

## DNA1 vs DNA2

Table 3. SPSS output of the reproducibility analysis using phage DNA isolated following two different protocols showing no significant difference between them (p = 0.080)

**Descriptive Statistics**

| | N | Mean | Std. Deviation | Minimum | Maximum |
|---|---|---|---|---|---|
| Results | 117 | 98.2128 | 2.93565 | 89.40 | 100.00 |
| Vars | 117 | 1.3932 | .49055 | 1.00 | 2.00 |

**Mann-Whitney Test**

**Ranks**

| | Vars | N | Mean Rank | Sum of Ranks |
|---|---|---|---|---|
| Results | DNA1 | 71 | 55.32 | 3927.50 |
| | DNA2 | 46 | 64.68 | 2975.50 |
| | Total | 117 | | |

**Test Statistics**

| | Values |
|---|---|
| Mann-Whitney U | 1371.500 |
| Wilcoxon W | 3927.500 |
| Z | -1.755 |
| Asymp. Sig. (2-tailed) | .079 |
| Exact Sig. (2-tailed) | .080 |
| Exact Sig. (1-tailed) | .039 |
| Point Probability | .000 |

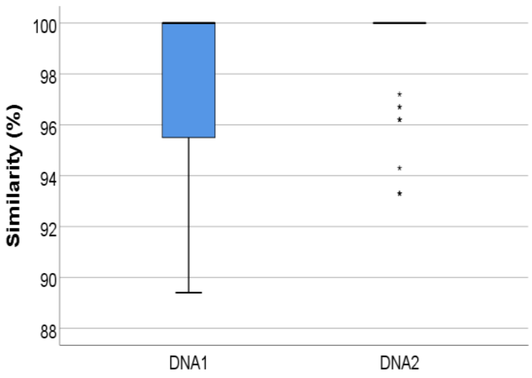


Figure 3. The plot illustrates 95% Confidence Intervals (CI) of dispersion estimates when using phage DNA amplified following two diffrent protocols

## Descriptives

| | Vars | | | Statistic | Std. Error |
|---|---|---|---|---|---|
| Results | DNA1 | Mean | | 97.7648 | .39211 |
| | | 95% Confidence Interval for Mean | Lower Bound | 96.9828 | |
| | | | Upper Bound | 98.5468 | |
| | | 5% Trimmed Mean | | 98.1013 | |
| | | Median | | 100.0000 | |
| | | Variance | | 10.916 | |
| | | Std. Deviation | | 3.30394 | |
| | | Minimum | | 89.40 | |
| | | Maximum | | 100.00 | |
| | | Range | | 10.60 | |
| | | Interquartile Range | | 4.50 | |
| | | Skewness | | -1.293 | .285 |
| | | Kurtosis | | .571 | .563 |
| | DNA2 | Mean | | 98.9043 | .31031 |
| | | 95% Confidence Interval for Mean | Lower Bound | 98.2794 | |
| | | | Upper Bound | 99.5293 | |
| | | 5% Trimmed Mean | | 99.1548 | |
| | | Median | | 100.0000 | |
| | | Variance | | 4.429 | |
| | | Std. Deviation | | 2.10459 | |
| | | Minimum | | 93.30 | |
| | | Maximum | | 100.00 | |
| | | Range | | 6.70 | |
| | | Interquartile Range | | .70 | |
| | | Skewness | | -1.708 | .350 |
| | | Kurtosis | | 1.616 | .688 |
