# Supplementary material for: Bacteriophage genotyping using BOXA repetitive-PCR
Source: BMC Microbiol. 2020 Jun 11;20:154. doi: 10.1186/s12866-020-01770-2 (PMC7291552; doi:10.1186/s12866-020-01770-2)
Supplement: Supplementary file 4 — Additional file 4 Influence of the propagating temperature and propagating host on the phage profile. This file provides the BOXA2R-PCRfingerprint profiles of lactococcal phages 712 and P087, which show the influence of the propagating temperature and their hosts on pattern reproducibility. [file 12866_2020_1770_MOESM4_ESM.pdf]

Additional file 4.

Influence of the propagation temperature on the phage profile

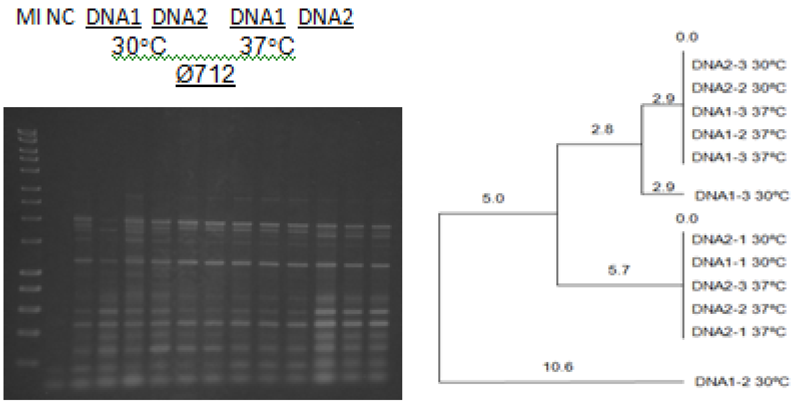

Figure A. Reproducibility testing of the BOXA2R-PCR using the DNA of the Ø712. The phage was propagated on *Lc. lactis* ssp *cremoris* C2 at 30°C and 37°C. The corresponding dendrogram was based on the UPGMA method. Three replicates per each DNA isolated by phenol-chloroform procedure (DNA1) and using the Qiagen kit (DNA2) were tested. M- HypperLadder I (Bioline). NC- negative control.

Influence of the propagating host on the phage profile

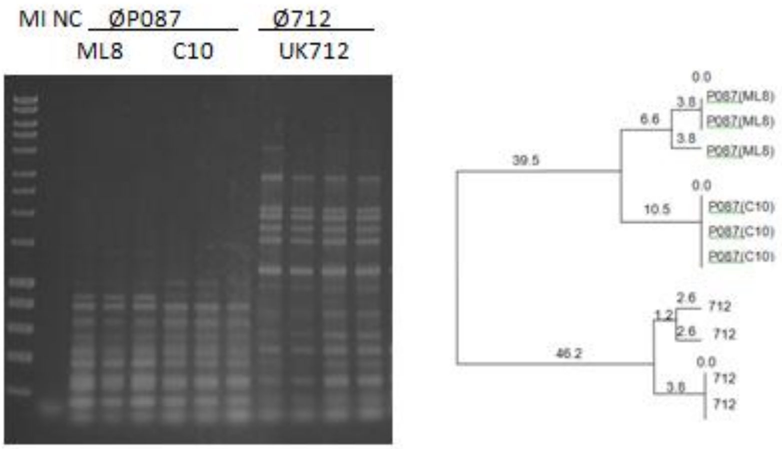

Figure B. Reproducibility testing of the BOXA2R-PCR. Three replicates of ØP087 DNA1 propagated on *Lc. lactis* ssp *lactis* biovar diacetylactis ML8 and *Lc. lactis* ssp *lactis* C10 at 30°C and four replicates of the Ø712 propagated on *Lc. lactis* ssp *cremoris* UK712 are displayed. The corresponding dendrogram was generated using the UPGMA method. M- HypperLadder I (Bioline). NC- negative control.
