# Supplementary material for: Bacteriophage genotyping using BOXA repetitive-PCR
Source: BMC Microbiol. 2020 Jun 11;20:154. doi: 10.1186/s12866-020-01770-2 (PMC7291552; doi:10.1186/s12866-020-01770-2)
Supplement: Supplementary file 5 — Additional file 5 Sequenced phage fragments. This file provides the list of sequenced phage fragments and details relating to their analysis, including; the primers used for the amplification of selected phage genes; results from the second round of PCR amplifications and BLAST analyses that used gene-specific primers; and a figure showing PCR amplifications of the selected phage DNA fragments using the forward and reverse primer pair corresponding to the annotated genes in each phage. [file 12866_2020_1770_MOESM5_ESM.pdf]

Additional file 5.

Sequenced phage fragments

Table 1. List of sequenced phage fragments and related information

FRAGMENT NAME	Band size- gel assessment (bp)	Band size- sequencing (bp)	UniProt KB annotation	Info	Amplification and sequencing primer
ØX174-1	650	637	P03631 **Replication-associated protein A (**Enterobacteria phage phiX174)[a]	E-value: 1.9e-91<br>Score: 745<br>Ident.: 89.1%	BOXA1R
ØX174-2	300	288	P03631-2 **Isoform A* of Replication-associated protein A** (Enterobacteria phage phiX174)[a]	E-value: 1.3e-3<br>Score: 108<br>Ident.: 55.3%	BOXA1R
ØLambda-1	450	480	P03689 **Replication protein P** (Escherichia phage lambda)[a]	E-value: 1e-15<br>Score: 166<br>Ident.: 43.2%	BOXA2R
ØT4-2	700	1089	A0A376YLU8 Putative baseplate structural protein (Escherichia coli)	E-value: 1.7e-13<br>Score: 188<br>Ident.: 36.7%	BOXA2R
ØDL4HV-1	800	881	U1P0V3 Phage integrase family (Halorubrum sp. J07HR59)	E-value: 1.6e-4<br>Score: 124<br>Ident.: 54.3%	BOXA1R
ØBU-1	400	354	A0A126HBL7 HNH endonuclease (Lactococcus phage 936 group)	E-value: 8.5e-23<br>Score: 201<br>Ident.: 69.0%	BOXA2R
Ø301-1	900	912	A0A1P8BM18 Baseplate/receptor binding protein (Lactococcus phage 98104)	E-value: 8.5e-28<br>Score: 198<br>Ident.: 61.8%	BOXA2R
Øc2-1	1200	1189	Q38305 Probable tape measure protein (Lactococcus phage c2)	E-value: 5.8e-17<br>Score: 145<br>Ident.: 66.7%	BOXA1R

a – Reviewed protein

Table 2. Primers used for the amplification of selected phage genes and results from the second round of PCR amplifications and BLAST analyses that used gene-specific primers

**Fragment**	**UniProt KB annotation (Top BLAST result)**	**Genome**	**CDS**	**Expected size (bp)**	**Primers for CDS amplification**	**PCR product size (bp)**	**BLAST results**
ØX174-1	P03631 Replication-associated protein A (Enterobacteria phage phiX174)a	J02482	AAA32570.1	1542	FWD: ATGGTTCGTTCTTATTACC<br>REV: TCATTTTCCGCCAGCA	~ 1500	E-value: 0.0<br>Score: 1819<br>Ident.: 93.30%
ØLambda-1	P03689 Replication protein P (Escherichia phage lambda)a	J02459	AAA96585.1	702	FWD: ATGAAAAACATCGCCGC<br>REV: TCATACACTTGCTCCTTTC	~ 700	E-value: 3.6e-156<br>Score: 1133<br>Ident.: 100.00%
ØT4-2	A0A376YLU8 Putative baseplate structural protein (Escherichia coli)	UGCD01000004	STK06109.1	648	FWD: ATGAGTAAAACAACACCGAC<br>REV: TTAGCCCATCGCCGAAT	~ 650	E-value: 3.9e-133<br>Score: 1004<br>Ident.: 98.50%
Øc2-1	Q38305 Probable tape measure protein (Lactococcus phage c2)	L48605	AAA92189.1	2121	FWD: ATGGCTAAAGAAAAATATGTC<br>REV: TCAACGCTTATTCAATTTAAC	~ 2000	E-value: 0.0<br>Score: 1529<br>Ident.: 94.70%

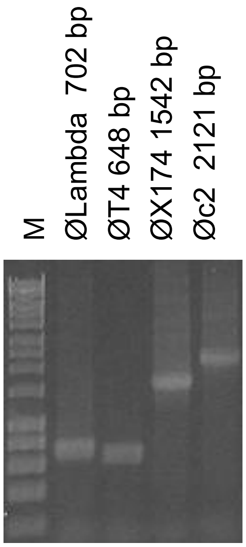

Figure 1. PCR amplifications of the selected phage DNA using the forward and reverse primer pair corresponding to the annotated genes in each phage. The correct product size was generated matching the targeted genes in: ØLambda (Replication protein P); ØT4 (Putative baseplate structural protein); ØX174 (Replication-associated protein A) and Øc2 (Probable tape measure protein)
