# Supplementary material for: Bacteriophage genotyping using BOXA repetitive-PCR
Source: BMC Microbiol. 2020 Jun 11;20:154. doi: 10.1186/s12866-020-01770-2 (PMC7291552; doi:10.1186/s12866-020-01770-2)
Supplement: Supplementary file 6 — Additional file 6 Sanger sequencing of the BOXA-PCR fragments. This file provides the alignment of the sequencing of the PCR products to the sequences of the corresponding proteins. [file 12866_2020_1770_MOESM6_ESM.pdf]

Additional file 6.

Sanger sequencing of the BOXA-PCR fragments

Figure 1. Alignment of the sequencing of the PCR product from ØX174 DNA to the sequence of the replication-associated protein A (Enterobacteria phage phiX174) (grey boxes indicate a match).

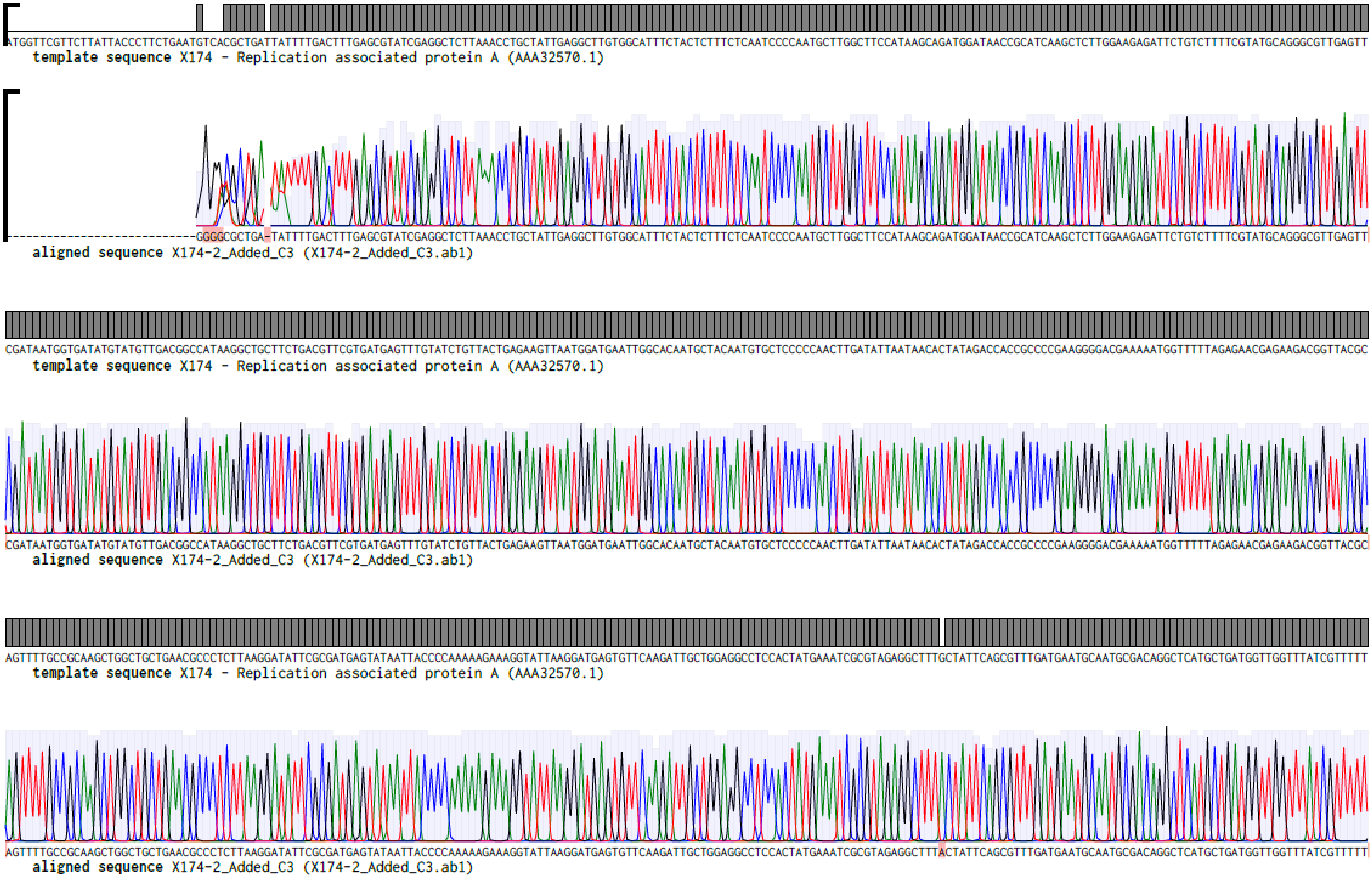

GACACTCTCACGTTGGCTGACGACCGATTAGAGGCGTTTTATGATAATCCCAATGCTTTGCGTGACTATTTTCGTGATATTGGTCGTATGGTTCTTGCTGCCGAGGGTCGCAAGGCTAATGATTCACACGCCGACTGCTATCAGTATTTTTGTGTGCCTGAGTATGGTACAGCTAATGGCCGTCTTCATTTCCATGCGGT

template sequence X174 - Replication associated protein A (AAA32570.1)

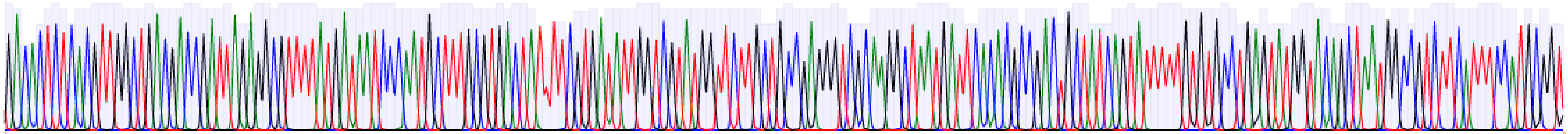

GACACTCTCACGTTGGCTGACGACCGATTAGAGGCGTTTTATGATAATCCCAATGCTTTGCGTGACTATTTTCGTGATATTGGTCGTATGGTTCTTGCTGCCGAGGGTCGCAAGGCTAATGATTCACACGCCGACTGCTATCAGTATTTTTGTGTGCCTGAGTATGGTACAGCTAATGGCCGTCTTCATTTCCATGCGGT

aligned sequence X174-2_Added_C3 (X174-2_Added_C3.ab1)

GCACTTTATGCGGACACTTCCTACAGGTAGCGTTGACCCTAATTTTGGTCGTCGGGTACGCAATCGCCGCCAGTTAAATAGCTTGCAAAATACGTGGCCTTATGGTTACAGTATGCCCATCGCAGTTCGCTACACGCAGGACGCTTTTTCACGTTCTGGTTGGTTGTGGCCTGTTGATGCTAAAGGTGAGCCGCTTAAAG

template sequence X174 - Replication associated protein A (AAA32570.1)

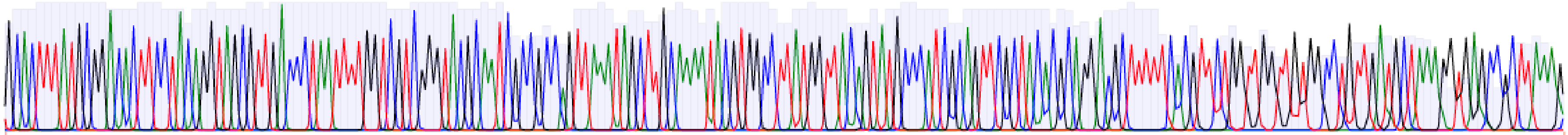

GCACTTTATGCGGACACTTCCTACAGGTAGCGTTGACCCTAATTTTGGTCGTCGGGTACGCAATCGCCGCCAGTTAAATAGCTTGCAAAATACGTGGCCTTATGGTTACAGTATGCCCATCGCAGTTCGCTACACGCAGGACGCTTTTTCACGTTCTGGTTGGTTGTGGCCTGTTGATGCTAAAGGTGAGCCGCTTAAAG

aligned sequence X174-2_Added_C3 (X174-2_Added_C3.ab1)

CTACCAGTTATATGGCTGTTGGTTTCTATGTGGCTAAATACGTTAACAAAAAGTCAGATATGGACCTTGCTGCTAAAGGTCTAGGAGCTAAAGAATGGAACAACTCACTAAAAACCAAGCTGTCGCTACTTCCCAAGAAGCTGTTCAGAATCAGAATGAGCCGCAACTTCGGGATGAAAATGCTCACAATGACAAATCTG

template sequence X174 - Replication associated protein A (AAA32570.1)

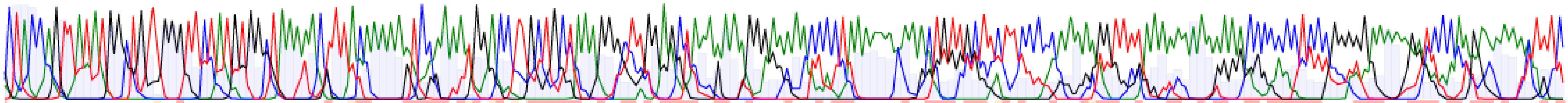

CTACCAGTTATATGGCTGTNGGGTTCTATGTGGCTAAATACTTTNCAAAAAGTCAGAAANGGNCCNTGCTGCTAAAGGGCTTGGAGCTAAAGAAAGGGAAGAACCCCTAAAAAACAAACTGTTGCTTNTTCCCCCAGAGNGGTTTTAGATACAAATAGACGCCACCTCCCGGGGGAGAAAGATCCCAAGACAAAAATGTC

aligned sequence X174-2_Added_C3 (X174-2_Added_C3.ab1)

TCCACGGAGTGCTTAATCCAACTTACCAAGCTGGGTTACGACGCGACGCCGTTCAACCAGATATTGAAGCAGAACGCAAAAAGAGAGATGAGATTGAGGCTGGGAAAAGTTACTGTAGCCGACGTTTTGGCGGCGCAACCTGTGACGACAAATCTGCTCAAATTTATGCGCGCTTCGATAAAAATGATTGGCGTATCCAA

template sequence X174 - Replication associated protein A (AAA32570.1)

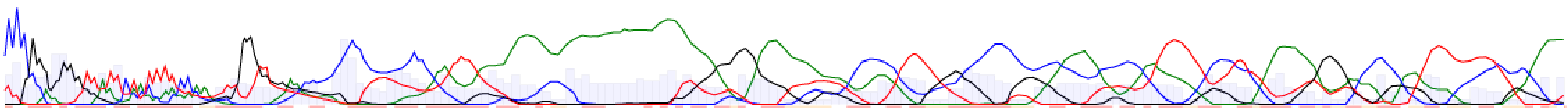

CCCGGAGGGGTTATTCTATTTTCCANNTGTGGTGGGAACCCCGCCCTTTCCCCCCCTTTTTAAAAAAAACNNAAAAAAAAAAAAAAAAAAAAAGNGGAAAANNNNNNAAACCACCCTTTNNNGGCCCCCCNNNGCCAAAAACCTCCCAATTTNNTCCCTTCNTAAAAAANANNACNNCCCANNTTTNNNTTCCNNNCCANA

aligned sequence X174-2_Added_C3 (X174-2_Added_C3.ab1)

CCTGCAGAGTTTTATCGCTTCCATGACGCAGAAGTTAACACTTTCGGATATTTCTGATGAGTCGAAAAATTATCTTGATAAAGCAGGAATTACTACTGCTTGTTTACGAATTAAATCGAAGTGGACTGCTGGCGGAAAATGA

template sequence X174 - Replication associated protein A (AAA32570.1)

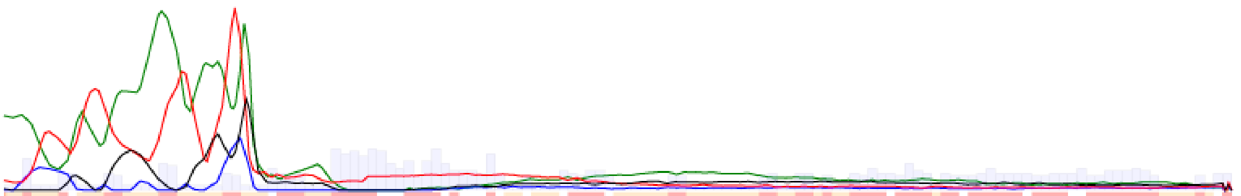

NNANNNTANNNAANNNNAANNNNNCTNNNATTGNNNTTTTTTTTTTTTTTTTTTTNNTNNNTTTTANNNNNNGANNNNNNNNGGNNCTNANNGGAAGAAAANAAAAAGGGAAGGAGGAGAGAAGAGNNNNCNGT

aligned sequence X174-2_Added_C3 (X174-2_Added_C3.ab1)

Figure 2. Alignment of the sequencing of the PCR product from ØLambda DNA to the sequence of the Replication protein P (*Escherichia phage lambda*) (grey boxes indicate a match).

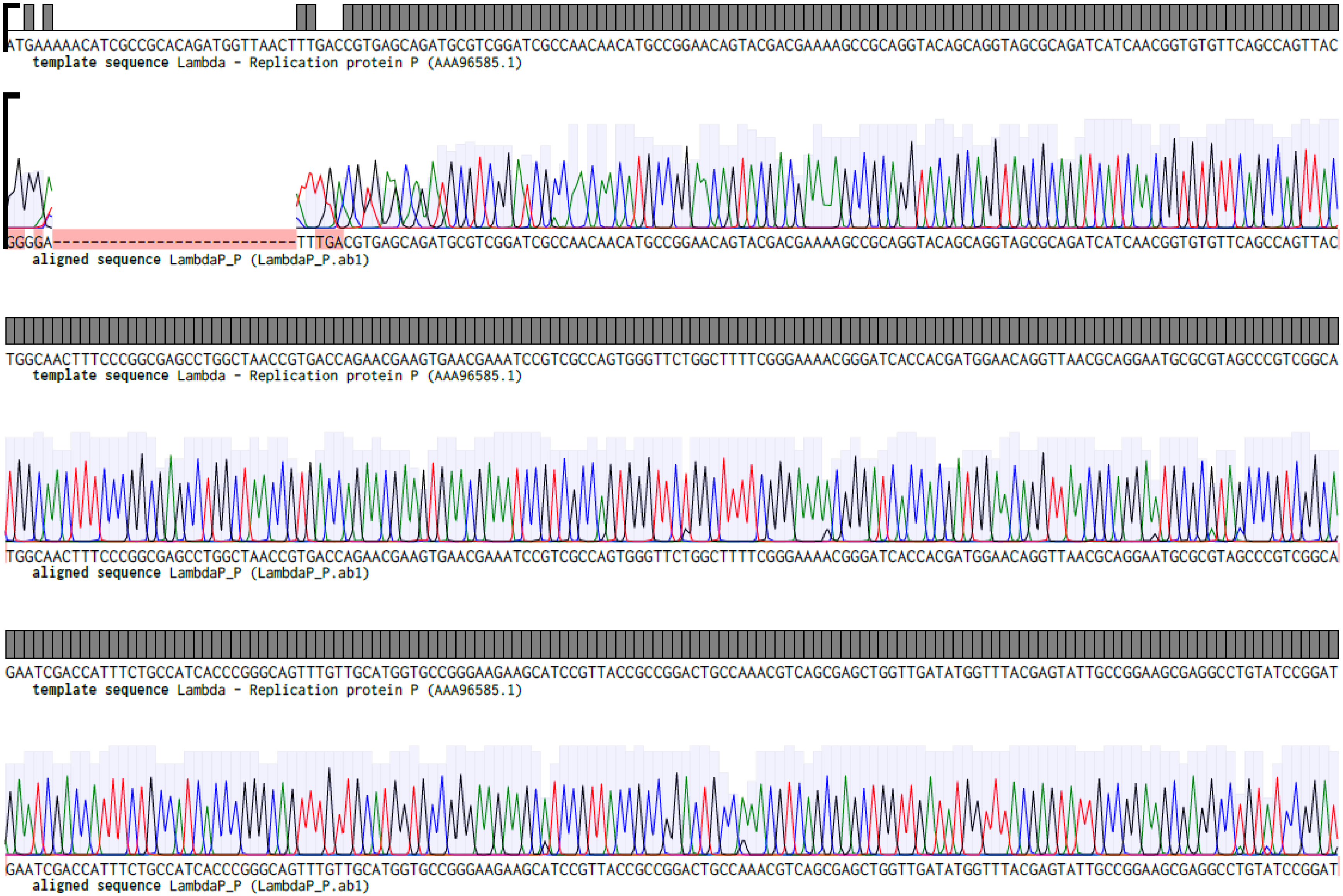

GCGGAGTCTTATCCGTGGAAATCAAACGCGCACTACTGGCTGGTTACCAACCTGTATCAGAACATGCGGGCCAATGCGCTTACTGATGCGGAATTACGCCGTAAGGCCGCAGATGAGCTTGTCCATATGACTGCGAGAATTA

template sequence Lambda - Replication protein P (AAA96585.1)

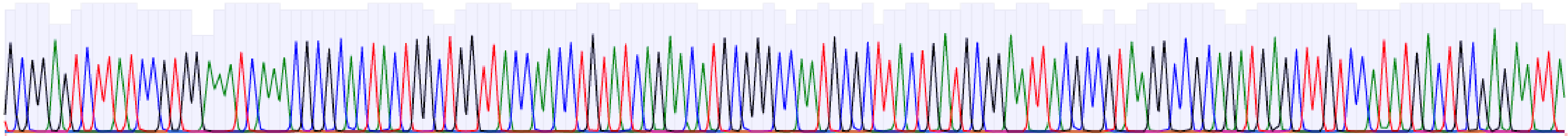

GCGGAGTCTTATCCGTGGAAATCAAACGCGCACTACTGGCTGGTTACCAACCTGTATCAGAACATGCGGGCCAATGCGCTTACTGATGCGGAATTACGCCGTAAGGCCGCAGATGAGCTTGTCCATATGACTGCGAGAATTA

aligned sequence LambdaP_P (LambdaP_P.ab1)

ACCGTGGTGAGGCGATCCCTGAACCAGTAAAACAACTTCCTGTCATGGGCGGTAGACCTCTAAATCGTGCACAGGCTCTGGCGAAGATCGCAGAAATCAAAGCTAAGTTCGGACTGAAAGGAGCAAGTGTATGA

template sequence Lambda - Replication protein P (AAA96585.1)

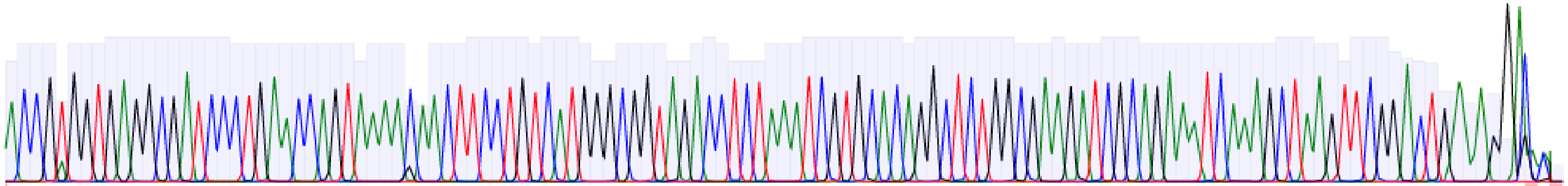

ACCGTGGTGAGGCGATCCCTGAACCAGTAAAACAACTTCCTGTCATGGGCGGTAGACCTCTAAATCGTGCACAGGCTCTGGCGAAGATCGCAGAAATCAAAGCTAAGTTCGGACTGAAAGGACCG

aligned sequence LambdaP_P (LambdaP_P.ab1)

Figure 3. Alignment of the sequencing of the PCR product from ØT4 DNA to the sequence of the putative baseplate structural protein (*Escherichia coli*) (grey boxes indicate a match).

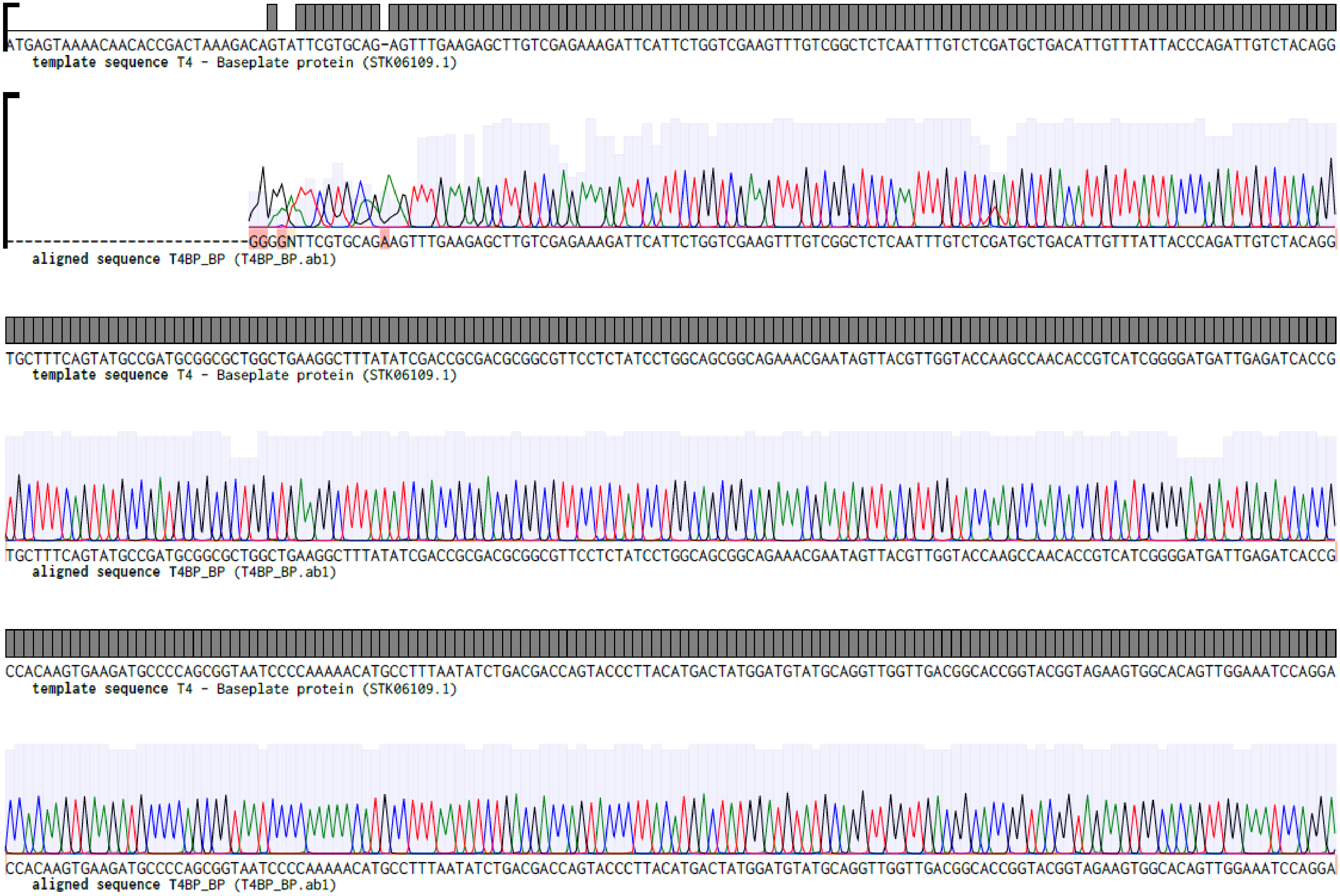

GGTGACATATACCGTTACGGCAGCCAAAGAATTTCTGGAAGTCGTGTTATCAAAGGCTCTTACTGCTGTCTGCTATAAGCTGGAAGTATTCGTGACGACCGATGGTAAGACCACGCAGTGGTCTTCCAGCACTATGTTCCGG

template sequence T4 - Baseplate protein (STK06109.1)

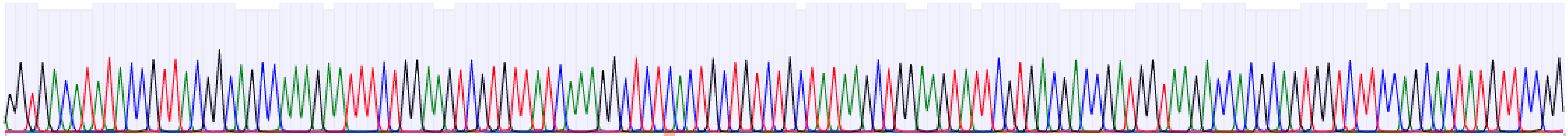

GGTGACATATACCGTTACGGCAGCCAAAGAATTTCTGGAAGTCGTGTTATCAAAGGCTCTCACTGCTGTCTGCTATAAGCTGGAAGTATTCGTGACGACCGATGGTAAGACCACGCAGTGGTCTTCCAGCACTATGTTCCGG

aligned sequence T4BP_BP (T4BP_BP.ab1)

TTAGCCGGTAGTAAAAGCCAGGTCTACGTTGAG-TTTATAAACCATCCGAGCAGTTGGGGGTTCGATTCGGCGATGGGCTAA----

template sequence T4 - Baseplate protein (STK06109.1)

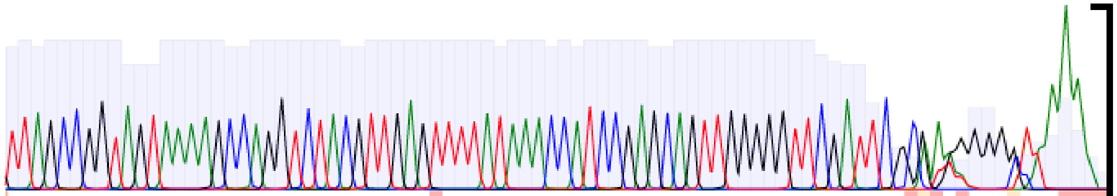

TTAGCCGGTAGTAAAAGCCAGGTCTACGTTGAGTTTTATAAACCATCCGAGCAGTTGGGGGTTCGATTCGCNAAGGGGNTAAAAAA

aligned sequence T4BP_BP (T4BP_BP.ab1)

Figure 4. Alignment of the sequencing of the PCR product from ØC2 to the sequence of the probable tape measure protein (*Lactococcus* phage c2) (grey boxes indicate a match).

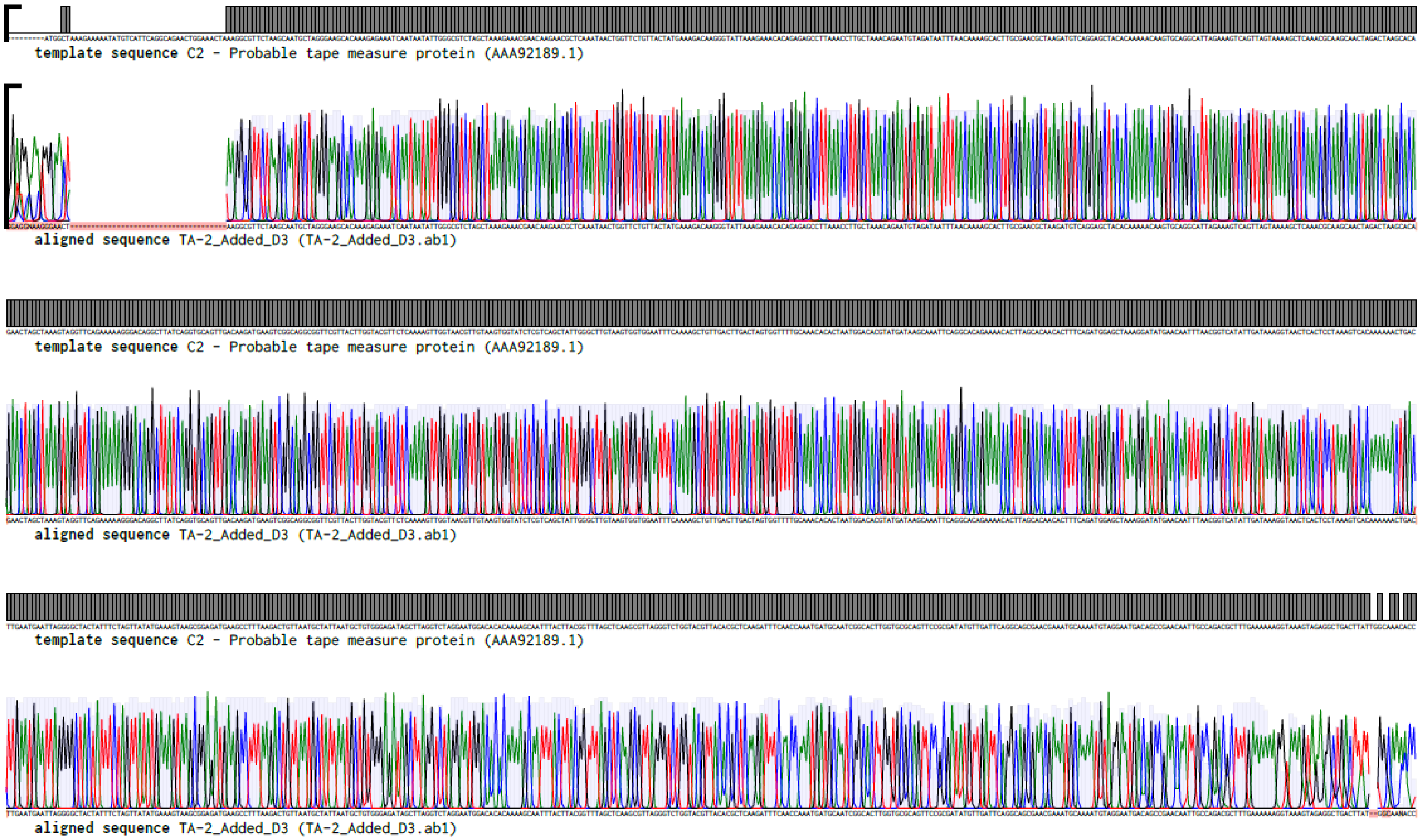

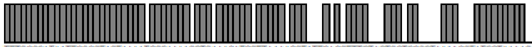

[illegible]

template sequence C2 - Probable tape measure protein (AAA92189.1)

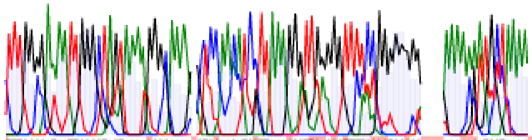

[illegible]

aligned sequence TA-2_Added_D3 (TA-2_Added_D3.ab1)

[illegible]

template sequence C2 - Probable tape measure protein (AAA92189.1)

aligned sequence TA-2_Added_D3 (TA-2_Added_D3.ab1)

[illegible]

template sequence C2 - Probable tape measure protein (AAA92189.1)

aligned sequence TA-2_Added_D3 (TA-2_Added_D3.ab1)

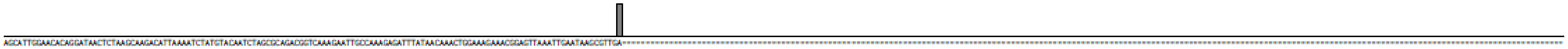

template sequence C2 - Probable tape measure protein (AAA92189.1)

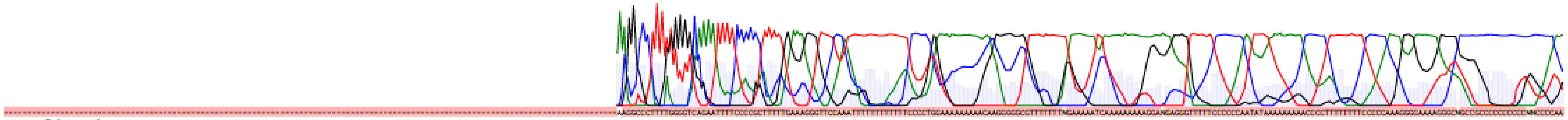

aligned sequence TA-2_Added_D3 (TA-2_Added_D3.ab1)

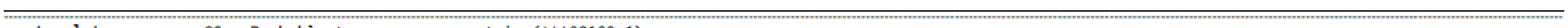

template sequence C2 - Probable tape measure protein (AAA92189.1)

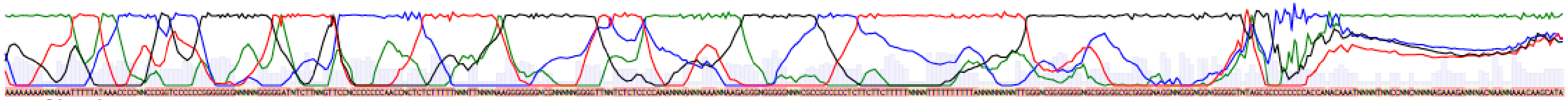

aligned sequence TA-2_Added_D3 (TA-2_Added_D3.ab1)

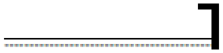

template sequence C2 - Probable tape measure protein (AAA92189.1)

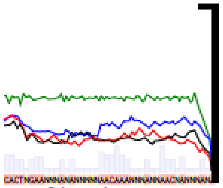

aligned sequence TA-2_Added_D3 (TA-2_Added_D3.ab1)
